# REGARDS: A Case Study in Aging and Disparities Research, Mentoring, and Data Sharing

**DOI:** 10.1093/geroni/igaa057.3150

**Published:** 2020-12-16

**Authors:** Virginia Howard, Jennifer Manly, Maria Glymour

## Abstract

Investigators in the NIH-funded REGARDS (REasons for Geographic and Racial Differences in Stroke) project have taken a novel approach to break the paradigm of epidemiologic studies limited to clinic-based convenience samples, by developing a national cohort of 30,239 black and white participants recruited from communities across all lower 48 US states, including 1,855 of the 3,033 counties. Mean age at enrollment (Jan 2003-Oct 2007) was 65.3 years. The four initial aims were to further understanding of: 1) geographic and racial differences in stroke risk factors; 2) geographic and racial differences in stroke incidence and mortality; 3) association of stroke risk factors and stroke risk (incidence and mortality) focusing on effect modification by race or region; and 4) establishment of a repository of serum, plasma, urine and DNA for use in future studies. When the grant was awarded, the study goals were broadened to include longitudinal remote assessment of cognitive function. A second in-home visit was completed May 2013-Dec 2016 including measures of functional status. The cohort is in its 17th year of follow-up. We will detail recruitment and enrollment methods, characteristics of the cohort and status, with brief overview of the biological, medical, psychosocial, environmental, and contextual data collected in the parent study. Speakers will discuss in more detail the stroke and cognitive data, ancillary studies focused on caregiver and heart disease outcomes, and provide examples of national and international mentoring that has leveraged REGARDS data. Finally, we will describe opportunities for additional data sharing and new ancillary studies.

